# Spatiotemporal Cluster Patterns of Hand, Foot, and Mouth Disease at the County Level in Mainland China, 2008-2012

**DOI:** 10.1371/journal.pone.0147532

**Published:** 2016-01-25

**Authors:** Chao Wang, Xia Li, Yingjie Zhang, Qin Xu, Fangfang Huang, Kai Cao, Lixin Tao, Jin Guo, Qi Gao, Wei Wang, Liqun Fang, Xiuhua Guo

**Affiliations:** 1 Department of Epidemiology and Health Statistics, School of Public Health, Capital Medical University, Beijing, China; 2 Beijing Municipal Key Laboratory of Clinical Epidemiology, Beijing, China; 3 Graduate Entry Medical School, University of Limerick, Limerick, Ireland; 4 National Center for Public Health, Surveillance and Information Services, Chinese Center for Disease Control and Prevention, Beijing, China; 5 School of Medical Sciences, Edith Cowan University, Perth, Western Australia, Australia; 6 State Key Laboratory of Pathogen and Biosecurity, Beijing Institute of Microbiology and Epidemiology, Beijing, China; Peking UIniversity, CHINA

## Abstract

**Background:**

Hand, foot, and mouth disease (HFMD) is known to be a highly contagious childhood illness. In recent years, the number of reported cases of HFMD has significantly increased in mainland China. This study aims at the epidemiological features, spatiotemporal patterns of HMFD at the county/district level in mainland China.

**Methods:**

Data on reported HFMD cases for each county from 1 January 2008 to 31 December 2012 were obtained from the Chinese Center for Disease Control and Prevention. Cluster analysis, spatial autocorrelation, and retrospective scan methods were used to explore the spatiotemporal patterns of the disease.

**Results:**

The annual incidences varied greatly among the counties, ranging from 0 to 74.31‰ with the median of 5.42‰ (interquartile range: 1.54‰–13.55‰) during 2008–2012 in mainland China. Counties close to provincial capital cities generally had higher incidences than rural counties. A seasonal distribution was observed between the northern and southern China, of which dual epidemic were shown in southern China and usually only one in northern China. Based on the global and local spatial autocorrelation analysis, we found that the spatial distribution of HFMD was presented a significant clustering pattern for each year (P<0.001), and hotspots of the disease were mostly distributed in coastal provinces of China. The retrospective scan statistic further identified the dynamics of spatiotemporal clustering areas of the disease, which were mainly distributed in the counties of eastern and southern China, as well as provincial capitals and their surrounding counties.

**Conclusions:**

The spatiotemporal clustering areas of the disease identified in this way were relatively stable, and imminent public health planning and resource allocation should be focused within those areas.

## Introduction

Hand, foot, and mouth disease (HFMD), predominantly caused by *enterovirus* (EV71) and *coxsackievirus* (CVA16), is a common infectious disease in children younger than 5 years of age. HFMD was first reported in New Zealand in 1957 [1]. In recent years, outbreaks of the disease have been reported frequently in most parts of the world, including the Asia-pacific region, especially in eastern and southeast Asia [2–5]. HFMD usually manifests with fever, a sore throat, rashes with blisters, and ulcerations on the hands, feet, legs or buttocks and mouth and may lead to severe complications including death due to the lack of specific treatments and vaccines to prevent the disease [6]. HFMD had the most reported cases, and the number of deaths ranked in the top five among all notifiable diseases since 2009 according to the Chinese Ministry of Health. More than 7.2 million cases and 2 457 fatal cases occurred between 2008 and 2012 [7]. Therefore, identifying the epidemiological characteristics of HFMD over space and time, along with hotspot locations, allows for early prevention and disease control.

Epidemiological studies of HFMD have indicated that diverse seasonal patterns of HFMD incidence were observed in southern and northern China [7–11]. Similarly, several spatial analyses have previously been conducted to describe spatial patterns, cluster locations and risk ratios of HFMD in some provinces in mainland China, such as Guangdong [12], Shandong [14], Beijing [15], Jiangsu [16], Guangxi [17] and Sichuan [18]. These studies mainly included a description of the epidemiological characteristics (demographic characteristics and temporal pattern), explorations of global or local spatial autocorrelations and detections of spatial or spatiotemporal clusters, and have specified the description of disease at the county level in different provinces. However few studies have been conducted at the county level throughout China. Identifying spatiotemporal patterns of HFMD might help determine the high-risk areas over time and ultimately guide public health interventions to control and prevent the disease. The aim of this study was to explore the spatiotemporal patterns and space-time clusters of HFMD at the county level across all of mainland China using surveillance data from 2008 to 2012 because a long-term, nationwide county-level HFMD study has been rarely reported.

## Materials and Methods

### Data Collection

All reported HFMD cases from 1 January 2008 to 31 December 2012 in mainland China were obtained from the National Notifiable Disease Surveillance System (NNDSS) of the Chinese Center for Disease Control and Prevention (Chinese CDC). The data included the monthly reported number of HFMD cases per county. The diagnostic criteria of HFMD were based on the clinical standard established by the National Health and Family Planning Commission of the People’s Republic of China in 2008, with or without fever, vesicular rash on the hands, feet, mouth, and buttocks. A confirmed case was defined based on laboratory evidence of enterovirus infection [19]. A total of 7 169 139 (99.6% of all cases included in the analysis by Xing [7]) geo-referenced cases at the county level were included in this study. Demographic data of each county were obtained from the National Bureau of Statistics of China [20].

Ethical approval and the consent from each individual subject was not required because the data were aggregated from the Chinese CDC.

### Cluster Analysis

Cluster analysis groups a set of objects into the same group (called a cluster) that are more similar to each other than other groups. This is the main task of an exploratory analysis and is also a common technique for statistical data analysis. Because counties with fewer cases (especially those with no cases in certain months) could not accurately describe the time characteristics of the region, the counties that had at least one cumulative case for the same month were categorized using SAS 9.2 (SAS Institute Inc., Cary, NC, USA) to identify different seasonal patterns of HFMD occurrence in China. A total of 2 108 counties were included in this analysis.

### Global and local spatial autocorrelation analysis

The autocorrelation statistic (Global Moran’s I) [21] was performed to detect the global spatial autocorrelation of HFMD autocorrelation across the settings. The significance of Moran’s I was assessed using Monte Carlo randomization. A statistically significant (P<0.05) estimate of Moran’s I indicates that neighboring counties have a similar incidence of HFMD and that the cases are likely to cluster at the county level in mainland China [14,22]

Local spatial autocorrelation analysis based on local indicators of spatial association (LISA) [23–24] was used to measure the local spatial autocorrelation of HFMD incidence at the county level in mainland China. LISA cluster and significance maps were used to detect local hot and cold spots of HFMD epidemics. The univariate LISA indicates the degree between the values of a given location and the average values of neighboring locations. Anselin local Moran’s I was used to determine whether there were positive spatial correlations (high-high clusters and low-low clusters) or negative spatial correlations (high-low clusters and low-high clusters). Spatial weights were based on the inverse distance weighted between polygon centroids that were constructed to identify spatial relationships between the counties. ArcGIS 10.1 (ESRI, Inc. Redlands, CA, USA) was used to perform this analysis.

### Retrospective space-time scan statistics analysis

The space-time scan statistic was defined by a cylindrical window using a circular geographic base with a height corresponding to time [12,25]. The base and height of the windows were varied to detect every possible spatiotemporal cluster. The center and the radius of the window change continuously according to the population of that area, and the height varies according to the likely cluster time. The null hypothesis of the model was that there was no difference between the incidence inside and outside the cylinder. For each scanning window, a likelihood ratio and relative risk were calculated to test the hypothesis. The window with the maximum likelihood was defined as the most likely cluster, and other windows with smaller likelihood that were also statistically significant (evaluated using Monte Carlo simulations) were defined as secondary clusters. The relative risk represents the aggregation risk of the cluster compared with the rest of the regions [26–30].

SaTScan^TM^ 9.0 software [http://www.satscan.org/] was used for the space-time analysis of HFMD. In this study, the spatial range of the space-time scan analysis included 2 922 counties in mainland China, and the time range were the 60 months from 2008 to 2012. We performed the analysis assuming a maximum cluster size of 10% of the total population and that clusters did not overlap with a previously reported cluster of HFMD, setting time as a default. The time frame of the scan analysis was set to be one year to control the time trends and observe the cluster changes across the whole study period [12].

Furthermore, all counties were divided into two categories based on whether they had been clustered or not. We calculated and compared the distance between each county’s centroid and the nearest provincial capital city using the Wilcoxon rank test in SAS 9.2 because the distributions of the distance were not normal.

## Results

**Fig 1:**
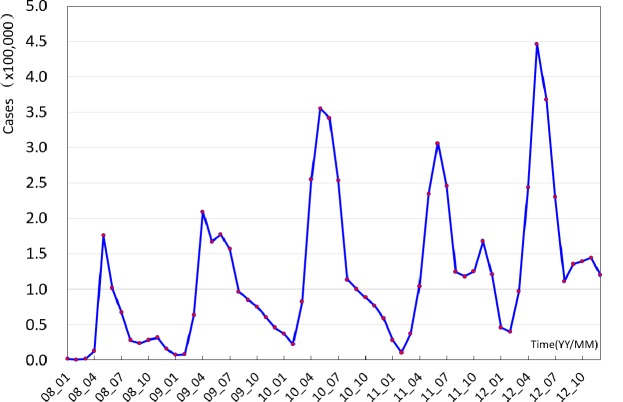
Monthly number of HFMD cases reported in mainland China, 2008–2012.

**Fig 2:**
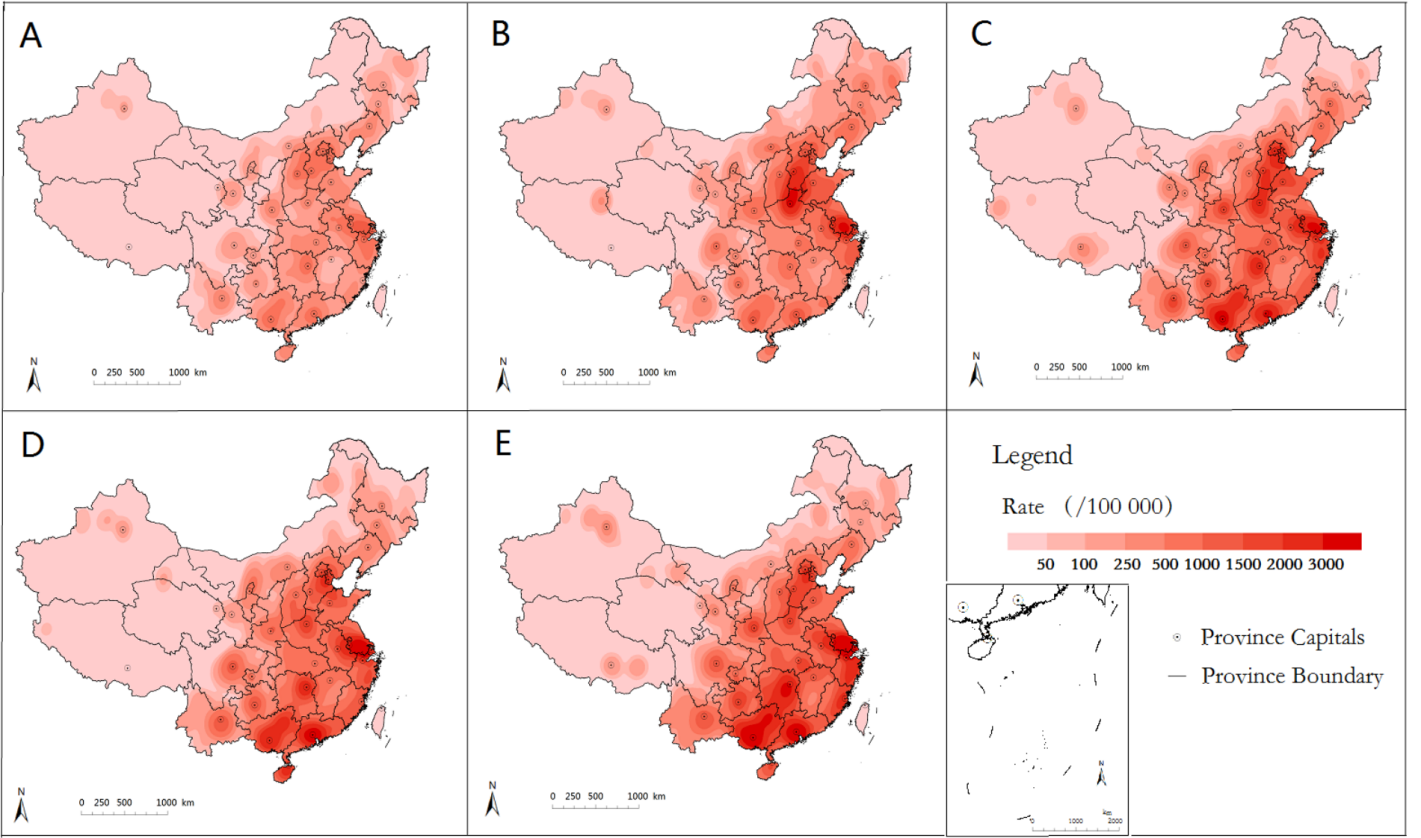
Yearly kernel density map of HFMD incidence in mainland China 2008–2012 (A-E).

A total of 7 169 139 HFMD cases were included in this study. The annual incidence of HFMD in counties varied greatly with a median of 5.42‰ (interquartile range: 1.54‰–13.55‰), ranging from 0 to 74.31‰ in mainland China. Fig 1 shows the epidemic trend of the monthly number of HFMD cases in mainland China. More cases occurred during April-July, indicating a potential seasonality of the incidence of HFMD. Fig 2 shows the spatial distribution of HFMD incidence across mainland China from 2008 to 2012. The incidences of HFMD varied greatly among the regions; higher incidence was shown in eastern and southern regions compared with western and northeastern China.

### Seasonal and Geographic Distribution of HFMD Cases

**Fig 3:**
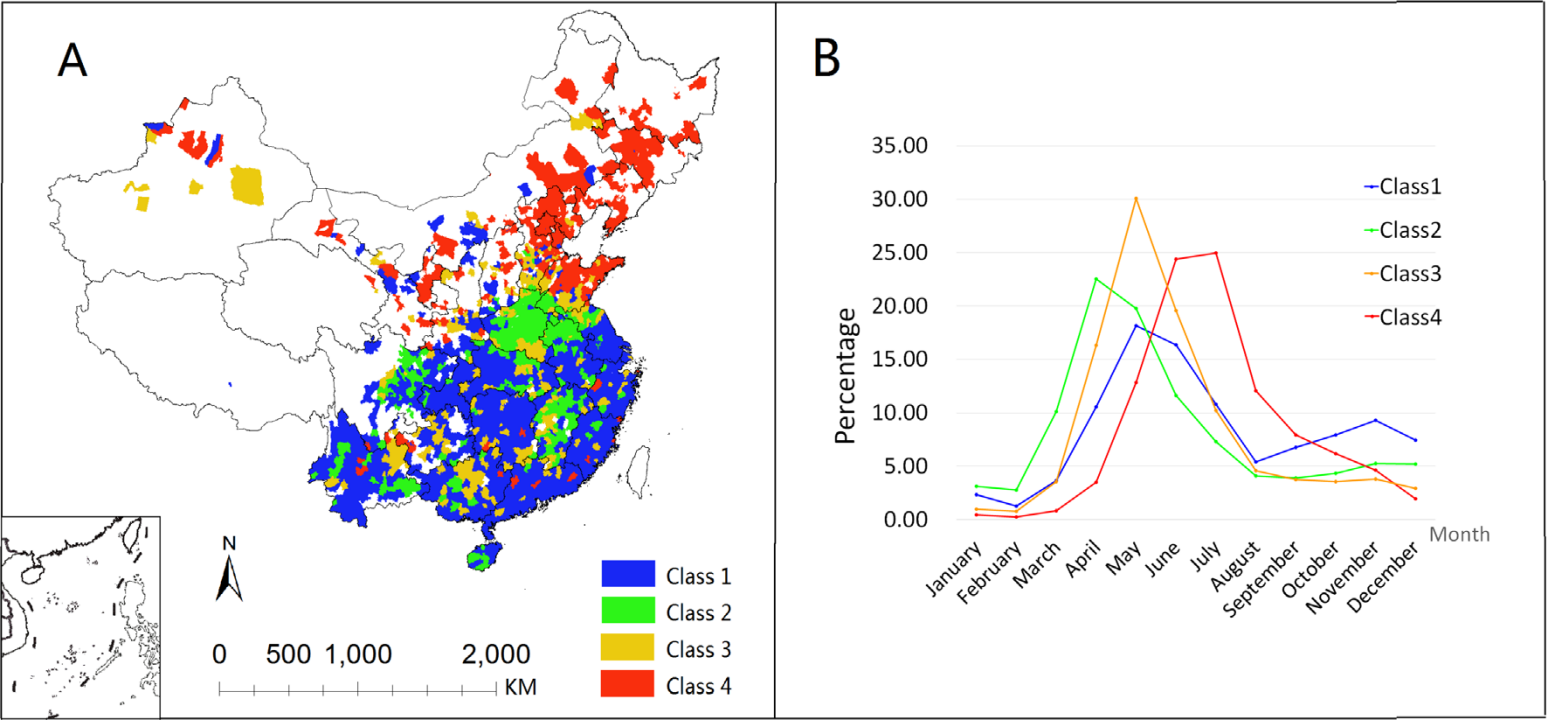
Locations (A) and time characteristics (B) of the four different categories based on the time distribution of every county with at least one monthly case. The line graph is the average of the counties under the same classification.

A total of 2 108 counties was included in the analysis and were further classified into four classes to explore seasonal patterns (Fig 3). The results of the cluster analysis showed that there were two epidemic peaks in Class 1 and Class 2 and only one in Class 3 and Class 4 ever year (Fig 3B). Fig 3A shows the distribution of the four classes. Class 1, which consisted of 1 050 counties, was mostly distributed in southern China (southern Jiangsu and Anhui, Zhejiang, Fujian, Guangdong, Guangxi, Hunan, Hubei, Yunnan and Hainan provinces), with a smaller epidemic peak during May—June and another larger peak during October—December compared with Class 2. Class 2 included 335 counties and was mainly distributed in the Henan, Jiangxi and northern Anhui provinces. This class was also scattered across the Sichuan, Yunnan, and Guangxi provinces with a main epidemic peak during April -May and a smaller epidemic peak during November—December. Class 3 (322 counties) was also mostly scattered across the country with a single epidemic peak in May. Class 4 (401 counties) was mostly distributed in northern China and also had a single epidemic peak during June -July.

### Spatial Autocorrelation Analysis

A high global spatial autocorrelation was found at the county level in mainland China from 2008 to 2012 (Moran’s I > 0.22, *P* < 0.001) (Table 1). This finding meant that the spatial distribution of HFMD was spatially aggregated in the whole country.

**Table 1:** Results of the spatial autocorrelation test on HFMD incidences at the county level in mainland China, 2008–2012.

Year	Moran’s I	Z-score	*P*-value
2008	0.24	56.6	<0.001
2009	0.23	56.3	<0.001
2010	0.23	54.1	<0.001
2011	0.25	59.6	<0.001
2012	0.22	54.8	<0.001

**Fig 4:**
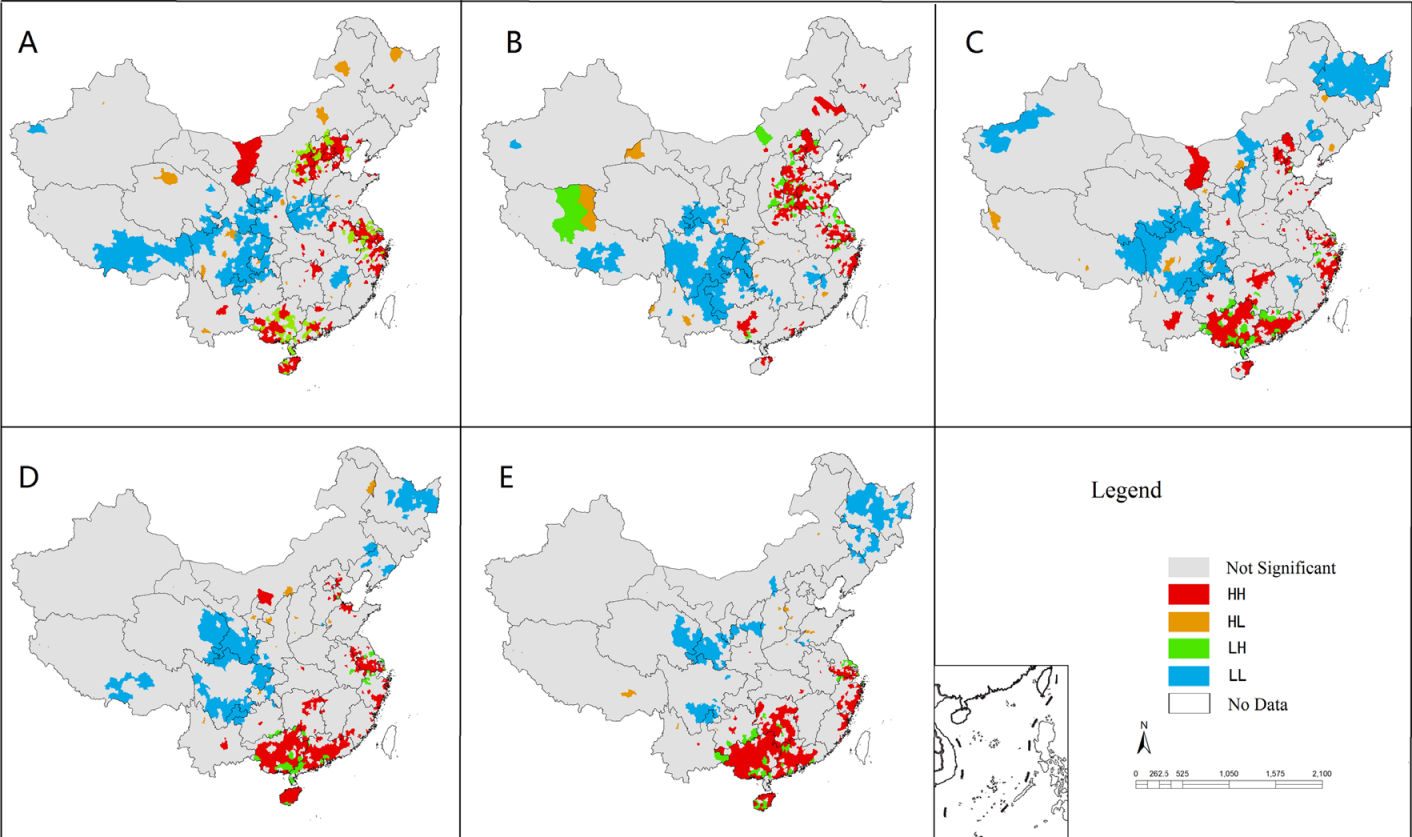
Spatial correlation cluster maps for HFMD incidence, 2008–2012 (A-E). HH, high-high cluster; HL, high-low cluster; LH, low-high cluster; LL, low-low cluster.

High-high and low-low clusters indicate the clustering of HFMD in a particular location with similar values. For example, a high-high cluster means that a county with high incidence is surrounded by counties with high incidence. Low-high and high-low clusters indicate spatial outliners that could be clues to unusual cases. In our study, lots of counties with a positive spatial association (high-high or low-low clusters) were identified across mainland China (Fig 4). High-high clusters were mainly distributed in the northern, eastern and southern provinces (Beijing, Hebei, Shanxi, Shandong, Henan, Jiangsu, Zhejiang, Hunan, Guangxi, Guangdong and Hainan), while low-low clusters were only found in inland provinces (Fig 4). A small number of counties were shown negative spatial associations (high-low or low-high clusters) were scattered across mainland China (Fig 4).

**Fig 5:**
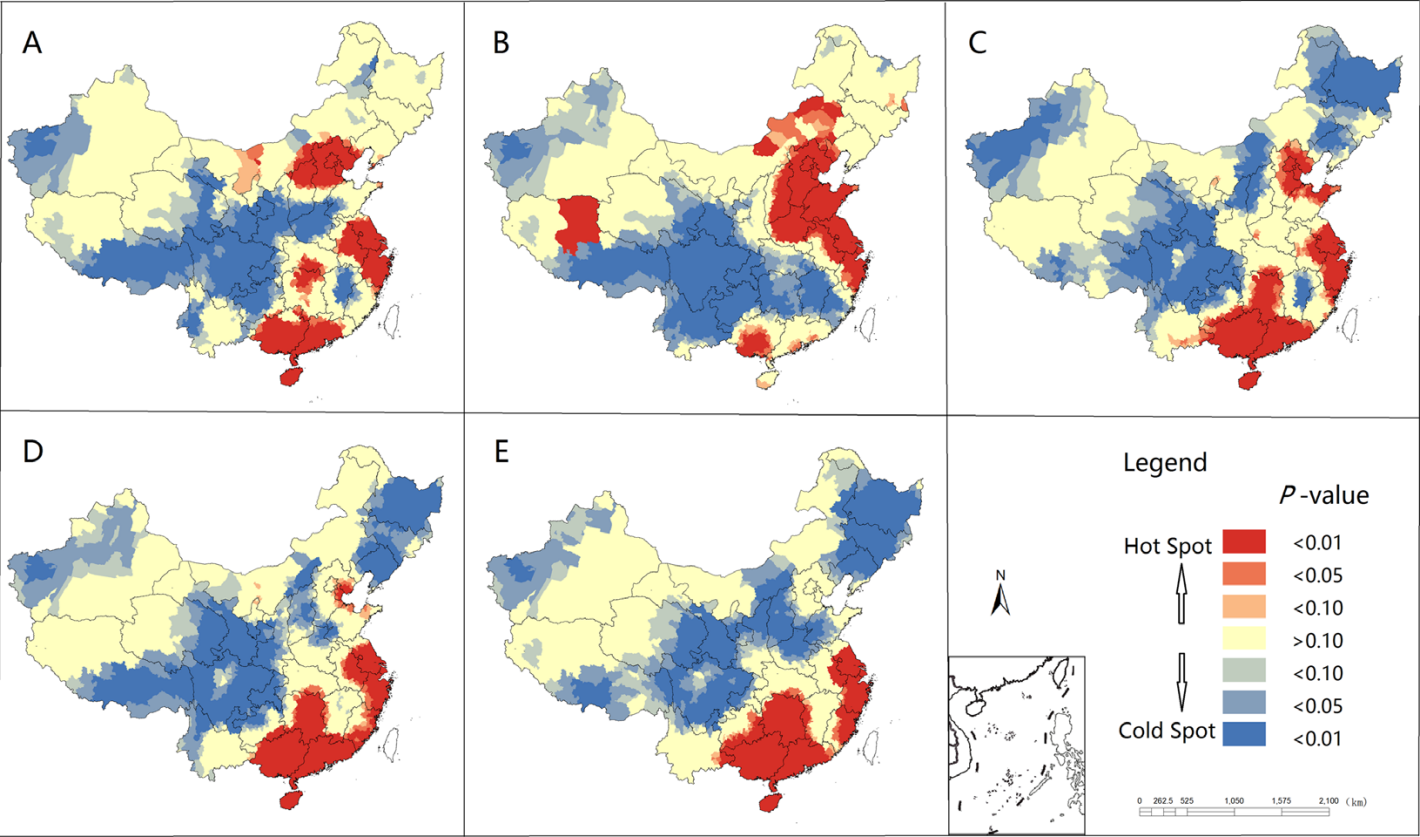
Local Indicators of Spatial Association (LISA) significance maps for the incidence of HFMD in mainland China, 2008 (A) to 2012 (E). Red indicates concentrated outbreaks, and blue indicates a low incidence.

The local hot spots and cold spots of HFMD epidemics were identified using the local indicators of spatial association (LISA) significance maps (a map of P values) in mainland China from 2008 to 2012 (Fig 5). The hot spots of HFMD were mainly distributed in the coastal or their surrounding provinces such as Beijing, Tianjin, Hebei, Shandong, Jiangsu, Zhejiang, Shanghai, Guangxi, Guangdong and Hainan. By contrast, the cold spots were located in inland provinces (Fig 5). The number and size of the hotspots changes variedly in each year, but their locations remained quite stable.

### Spatiotemporal Clusters

**Fig 6:**
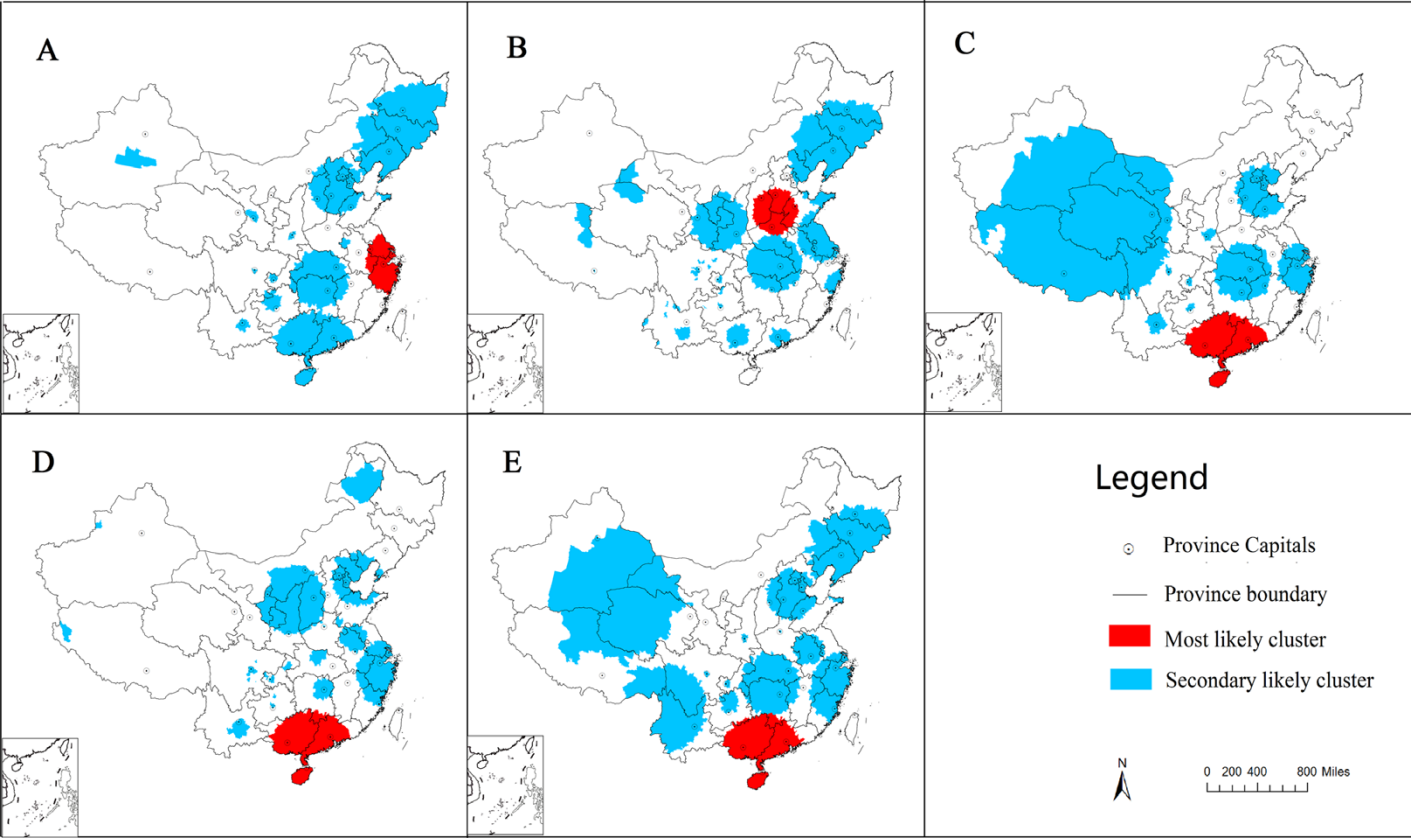
Spatiotemporal clusters of HFMD in mainland China, 2008–2012(A-E).

Spatiotemporal clusters were identified using space-time scan statistics method. At least 10 clusters were found each year during the study period (Fig 6). The centroid of the most likely cluster in 2008 was located at the Zhejiang province 30.32N, 122.25E, of which the radius was 402.95 km (including 208 counties in Zhejiang, southern Jiangsu, and southeast Anhui provinces). The relative risk of the cluster was 6.17, and the cluster time was from 1 May to 31 July 2008. In 2009, the most likely cluster was observed in the Shandong, Hebei, Henan, and Shanxi provinces including 281 counties. The relative risk of the cluster was 4.55, and the cluster time was from 1 April to 31 July 2009. The most likely clusters during 2010–2012 were located in more than 220 counties in southern China (Guangdong, Guangxi, and Hainan provinces). The relative risks ranged from 4.38 to 4.50, and the cluster time lasted from October 2011 to September 2012 (Table 2). All secondary clusters of HFMD with relative risks are scattered in Fig 6A–6E and shown in S1 Table.

**Table 2:** The most likely space-time clusters of HFMD in mainland China, 2008–2012.

Scan Timeframe	Cluster Time	Centroid (latitude, longitude)/ Radius(km)	Cluster Counties	Relative Risk	*P*-value
2008	May 1–Jul 31	(30.32N, 122.25E)/402.95	208	6.17	<0.001
2009	Apr 1–Jul 31	(36.39N, 114.24E)/267.61	281	4.55	<0.001
2010	Apr 1–Jul 31	(21.44N, 110.70E)/513.78	226	4.50	<0.001
2011	May 1–Oct 31	(21.57N, 110.97E)/494.26	225	4.45	<0.001
2012	Apr 1–Sep 30	(21.84N, 110.53E)/487.49	235	4.38	<0.001

The Wilcoxon rank test was applied to compare the difference of median distance to the nearest provincial city between clustered counties and non-clustered counties and showed that the clustered counties were closer to the provincial capital cities (Table 3).

**Table 3:** Distance from the provincial capital city of clustered counties and non-clustered counties, 2008–2012.

Year	No. of clustered counties	Distance for non-clustered counties(km)^a^	Distance for clustered counties(km)^a^	*P-*value^*b*^
2008	1 337	185.02 (118.10,264.21)	126.09 (68.12, 199.39)	<0.001
2009	1 320	188.38 (120.02,280.00)	125.49 (71.73,194.15)	<0.001
2010	1 227	183.11 (119.88,262.65)	122.34 (67.44,199.31)	<0.001
2011	1 120	179.12 (110.71,275.94)	126.68 (71.04,193.03)	<0.001
2012	1 556	181.72 (117.15,258.92)	135.02 (72.09,135.02)	<0.001

^*a*^ Average distance to the nearest provincial capital city from each county

^b^ Wilcoxon rank test.

**Fig 7:**
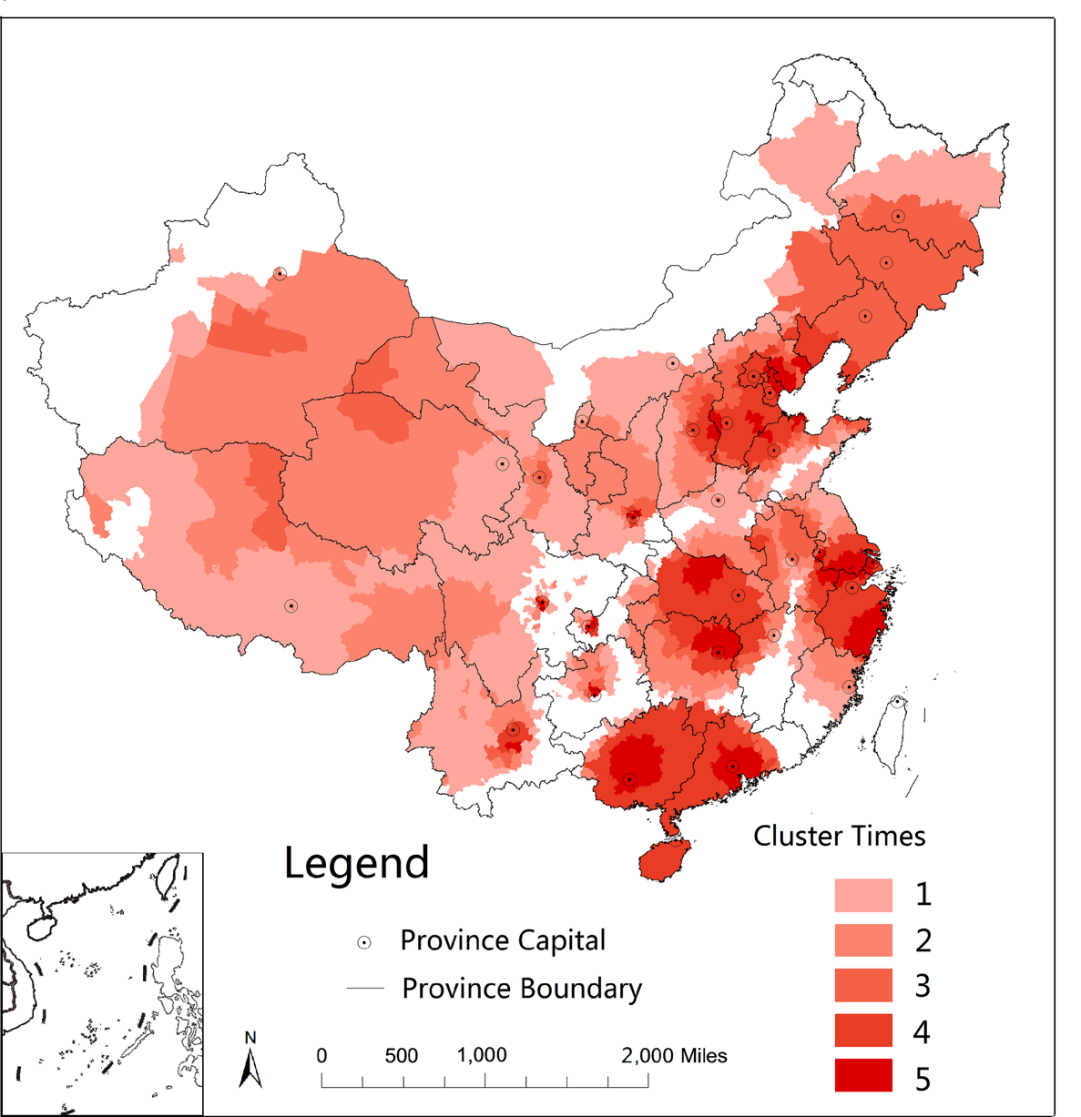
Detected times of spatiotemporal clusters of HFMD in mainland China, 2008–2012.

The characteristics of the spatiotemporal cluster patterns of HFMD were summarized in Fig 7. Our study observed four larger and several smaller clusters. Most clusters were distributed in higher population density areas along with high socioeconomic prominent locations than those outside cluster areas.

## Discussion

This study analyzed the spatial epidemiological characteristics of HFMD at the county level from 2008 to 2012 across mainland China. We explored the differences in seasonal characteristics between the northern and southern regions of China.

Our study showed that the annual average incidence of HFMD varied from year to year in each county in China. The average incidence of HFMD was below 0.1/1 000 population in most counties of western China and was found to be 10 times higher in the counties in eastern and southern China. However, the incidence of HFMD varied greatly between the regions, and a rising trend was observed in recent years (Fig 1), indicating that more effort is needed to prevent and control the disease in those high incidence regions.

A different seasonal pattern of HFMD was observed in mainland China. Hu predicted the incidence of HFMD using multiple seasonal autoregressive integrated moving average (ARIMA) models and found that the prediction was improved after categorizing the provinces into two strata using HFMD incidence patterns [31]. In this study, there were two seasonal peaks (the first peak was from April to June, and the second peak was in November) in southern China similar to other studies, including Singapore [32], Malaysia [33], Hong Kong [34], and Taiwan [35]. However, only one peak at the end of spring and beginning of summer (from May to July) was found in northern China and occurred nearly 1–2 months later after the first seasonal peak in southern China. Notably, not all of the counties strictly adhered to this rule even in northern (or southern) China, and the seasonal characteristics of the disease did not agree with each other (Fig 3). Different climatic conditions may lead to different seasonal characteristics between the northern and southern regions. Furthermore, the temperature, humidity, including other meteorological indicators and people’s living standards may also affect the disease epidemics in the different regions. Therefore, future studies should be considered by including those indicators.

This study observed the global spatial autocorrelation at the district/county level in China, which was a similar finding revealed at the provincial level by Xiao [36]. LISA cluster maps, hotspots, and spatiotemporal analysis with scan statistics found that the high HMFD incidence areas in Beijing, Tianjin, Hebei, Shandong, Henan, Jiangsu, Shanghai, Zhejiang, Guangdong, Guangxi, and Hainan provinces in China possess a higher population density along with the major transient areas. In addition, compared with studies by province [12–17], we found that many clusters were not only distributed within a province but also crossed to various provinces, indicating the importance of cooperation between provinces to prevent and control disease transmission.

This study also suggested that the distance from a provincial capital city affects the aggregation of HFMD. One explanation could be that counties with a shorter distance to provincial capitals in which traffic flows easily with a large transient population (e.g., administrative capitals, urbanization as well as being a business hub) are more prone to being a disease cluster. However, the development of the health care system may be slower. High population densities and poor health care might be the cause of the high incidence of the disease.

Due to the varying time and spatial characteristics of HFMD, different measures and precautions should be based on the various times and regions. First, greater emphasis should be placed on areas of eastern and southern China, especially in peak season (from April to July) and in counties near provincial capitals. Unified management of disease control at the national level and cooperation between provinces and regions is essential because many clusters cross multiple provinces. Considering that HFMD is showing an increasing trend, more effort is required for its prevention and control.

There are some limitations associated with this study. First, HFMD has been listed as a notifiable infectious disease since 2 May 2008. Consequently, we could not assure the accuracy of the relatively new surveillance monitoring system because some mild cases might be missed or may not be reported [7]. In this regard, patient information might be lost that could potentially cause geographical heterogeneity with the reporting system of cases [37–38]. In this study, we used the crude incidence rate instead of the incidence rate in children because we could not obtain child population data for each county. This limitation may affect our conclusions about disease prevention and control among children especially because they account for 90% of cases. Another limitation is with respect to the study methods. The space-time cylinder methods used to detect the clusters differed from the actual polygons (replaced by the centroid); the study results may have been affected by this difference. Several methods (Moran’s I, Geary’s C, etc.) are available to detect global spatial autocorrelation [39–40], and we used the most common one (Moran’s I) in this study. Lastly, we selected the county as the spatial scale, and more precise results may have been attained if a smaller spatial scale had been used for the study analysis.

## Conclusion

In conclusion, HFMD is a common infectious disease in China, and children under 5 years of age are more vulnerable. This study highlighted the epidemic characteristics and spatiotemporal clusters of HFMD at the county level from 2008 to 2012 in mainland China. We explored differences in seasonal characteristics between the northern and southern regions of China in which the seasonal peak began about a month later in the northern region than that in the southern regions. In addition, the southern regions had a second peak in the autumn. The government and stakeholders in those areas should be more aware about HFMD and pay more attention and provide appropriate public health measures to maximize cost-effectiveness, management and prevention of the disease. Nationally, the prevalence of HFMD has been primarily located in the eastern and southern provinces of China, indicating that more focus is needed in eastern and southern China. We also found that spatiotemporal clusters were not only distributed within a certain province but also crossed the various provinces. These findings show that cooperation between each province is important to prevent and control hand, foot and mouth disease.

## Supporting Information

S1 Table The secondary likely space-time clusters of HFMD in mainland China, 2008–2012. (XLSX)
